# Worldwide Evaluations of Quinoa—Biodiversity and Food Security under Climate Change Pressures: Advances and Perspectives

**DOI:** 10.3390/plants12040868

**Published:** 2023-02-15

**Authors:** Cataldo Pulvento, Didier Bazile

**Affiliations:** 1 Department of Soil, Plant and Food Science (DISSPA) University of Bari, 70121 Bari, Italy; 2 CIRAD, UMR SENS, F-34398 Montpellier, France; 3 SENS, Univ Montpellier, CIRAD, F-34398 Montpellier, France

## 1. Introduction

Quinoa (*Chenopodium quinoa* Willd.) is an Andean herbaceous crop that has attracted increasing interest in recent years thanks to its ecophysiological behavior and the nutritional characteristics of its seeds. The quinoa boom followed the celebration of the International Year of Quinoa in 2013 by the United Nations (FAO), when numerous initiatives were implemented to spread the positive characteristics that make quinoa a suitable crop with which to fight world hunger. In this Special Issue, we wanted to summarize the state of the art and the main research activities that are currently underway in different parts of the world.

## 2. Ecophysiological Traits and Adaptability

A bibliographic analysis of selected papers published from 2000 to 2020 highlighted that the number of studies on the best agronomic practices for quinoa strongly increased after 2013, when the FAO celebrated the International Year of Quinoa and disseminated the importance of quinoa as a high-quality protein crop resistant to unfavorable environments. Experimentation activity especially increased in countries characterized by a hot, arid climate and water scarcity (Morocco, Egypt, Burkina Faso, and the UAE), as well as in countries at risk of water and salt stress due to climate change (Italy, Greece, Turkey, Pakistan, and the USA), with trials focused on the effect of deficit irrigation and the use of saline water on quinoa yield and quality [1]. The same theme was also taken up by the papers published in this Special Issue; quinoa confirmed its adaptability to arid environments such as the Brazilian Cerrado, where water regimes between 309 and 389 mm do not reduce grain yield with respect to higher irrigation volumes [2].

In the same way, a field experiment in the southern Atacama Desert in Chile to investigate the responses to reduced irrigation of nine previously selected coastal lowland self-pollinated (CLS) lines and the commercial cultivar Regalona showed that several lines performed best when faced with a 50% reduction in irrigation [3].

Bharami et al. [4] studied the yield response of quinoa cv. Titicaca under field conditions in Iran and showed that the application of 75% of full irrigation requirements led to NO_3_-N accumulation in upper soil layers, thus facilitating nitrogen uptake and reduced nitrate loss to deeper layers of the soil and allowing for a reduction in the optimal nitrogen fertilization level for the study area.

Quinoa responds positively to fertilization in the Bolivian Altiplano [5], with differences among irrigated and rainfed conditions; quinoa can produce 1850 kg grains ha^−1^ with 50 kg N ha^−1^ under irrigated conditions and 670 kg grains ha^−1^ with 15 kg N ha^−1^ in rainfed conditions.

Rehman et al. [6] demonstrated, in Pakistan, that urea enriched with urease and nitrification inhibitors simultaneously can be used to improve the N uptake, seed yield, and grain protein contents in quinoa.

Quinoa was confirmed as maybe being a complementary crop in the marginal lands of high salinity in Egypt and the Mediterranean region [7]. Delatorre-Herrera et al. [8] demonstrated that the salinity tolerance of salares ecotypes is due to their ability to control non-diffusional components, indicating their superior photosynthetic capacity under salt stress conditions. Quinoa has also been proven to be a promising crop in cases of heat stress, with increased values of crude protein, ash, phosphorus, calcium, and relative feed [9].

Many papers from the literature are focused on the study of the best time and density for sowing, which represent the main agronomic practices for the introduction of a crop to a new environment [1]. In this Special Issue, new field trials evaluated the adaptability of quinoa to new environments in terms of yield, quality, and physiochemical characteristics in Belgium [10,11], Morocco [12], Pakistan [13], and Spain [14], in addition to selecting promising materials for breeding programs under greenhouse conditions [15]. The cultivation of quinoa was also reviewed in Pakistan [16] and Ecuador [17]. A large group of researchers from universities and research institutes from all over the world have proposed standard methodology guidelines [18] to be used for the phenotypic characterization of quinoa in order to improve comparability among field trials across the globe and to facilitate collaborations with the Global Collaborative Network on Quinoa (gcn-quinoa.org). Aspects related to quinoa diseases were reviewed by Colque-Little et al. [19], who summarized existing information on symptoms and causal agents. In Central Italy, the presence of *P. variabilis* and *F. equiseti* was monitored on *C. quinoa* [20]. Seed dormancy and breeding as well as nonbreeding strategies for enhancing resistance to preharvest sprouting in quinoa were reviewed by McGynti et al. [21].

Other ecological aspects, such as the geographical distribution of herbivore arthropods that affect the production of quinoa [22] and the impact of insecticides on insect pests of quinoa, as well as their side effects on the arthropod community [23], were analyzed in Chile and Peru.

## 3. Quinoa Seed Quality and Post-Harvest Activities

Hussain et al. [24] summarized recent findings regarding the nutritional and phytochemical properties of quinoa grains. A spectroscopy analysis of different quinoa cultivars grown under greenhouse conditions was conducted by García-Parra et al. [25] to evaluate the structural characterization and antioxidant capacity of quinoa.

The profiles of bioactive compounds in seeds of two quinoa varieties, Regalona-Baer and Titicaca, grown in Northern Italy, compared to that of seeds of those varieties grown in Chile and Denmark, were respectively assessed in order to establish the best conditions (genotype/geographical cultivation zone) leading to seed enrichment in functional compounds [26].

The pearling of quinoa seeds, nutrients, and saponin contents was evaluated to determine a correct standard for postharvest seed processing [27]; the description of a project aimed at the development of a potential quinoa value chain in order to improve food and nutritional security in rural communities in Rehamna, Morocco, was also reported [28].
